# A Novel *Fiber-1-*Edited and Highly Attenuated Recombinant Serotype 4 Fowl Adenovirus Confers Efficient Protection Against Lethal Challenge

**DOI:** 10.3389/fvets.2021.759418

**Published:** 2021-11-22

**Authors:** Yaru Mu, Quan Xie, Weikang Wang, Hao Lu, Mingjun Lian, Wei Gao, Tuofan Li, Zhimin Wan, Hongxia Shao, Aijian Qin, Jianqiang Ye

**Affiliations:** ^1^Ministry of Education Key Laboratory for Avian Preventive Medicine, Key Laboratory of Jiangsu Preventive Veterinary Medicine, Yangzhou University, Yangzhou, China; ^2^Jiangsu Co-innovation Center for Prevention and Control of Important Animal Infectious Diseases and Zoonoses, Yangzhou, China; ^3^Institutes of Agricultural Science and Technology Development, Yangzhou University, Yangzhou, China; ^4^Joint International Research Laboratory of Agriculture and Agri-Product Safety, the Ministry of Education of China, Yangzhou University, Yangzhou, China

**Keywords:** FAdV-4, *fiber-1*, CRISPR/Cas9, recombinant virus, attenuation, protection, vaccine

## Abstract

Currently, a fatal disease of hepatitis-hydropericardium syndrome (HHS) caused by serotype 4 fowl adenovirus (FAdV-4) has spread worldwide and resulted in tremendous economic losses to the poultry industry. Various vaccines against FAdV-4 were developed to control the disease; however, few live-attenuated vaccines were available. In this study, we targeted the N-terminal of *fiber-1* and rescued a recombinant virus FAdV4-RFP_F1 expressing the fusion protein of RFP and Fiber-1 based on the CRISPR/Cas9 technique. *In vitro* studies showed that FAdV4-RFP_F1 replicated slower than the wild type FAdV-4, but the peak viral titer of FAdV4-RFP_F1 could still reach 10^7.0^ TCID_50_/ml with high stability in LMH cells. Animal studies found that FAdV4-RFP_F1 not only was highly attenuated to the 2-week-old SPF chickens, but could also provide efficient protection against lethal challenge of FAdV-4. All these demonstrate that the recombinant virus FAdV4-RFP_F1 could be as an efficient live-attenuated vaccine candidate for FAdV-4, and the N-terminal of *fiber-1* could be as a potential insertion site for expressing foreign genes to develop FAdV-4-based vaccine.

## Introduction

Fowl adenovirus (FAdV) belongs to genus *Aviadenovirus*, family *Adenoviridae* (1). FAdV is a kind of non-enveloped viruses with a double-stranded DNA genome, clustered into 5 species (FAdV-A to FAdV-E) with 12 serotypes (FAdV-1 to FAdV-8a, FAdV-8b, and FAdV-9 to FAdV-11) based on the restriction enzyme digestion profile and sera cross-neutralization assay (2). FAdV can infect chickens through both vertical propagation and horizontal transmission. Of these 12 serotypes of FAdV, FAdV-4 is thought to be a highly pathogenic pathogen for poultry. The infection of FAdV-4 mainly results in hepatitis-hydropericardium syndrome (HHS) with a short latency, sudden onset, and high mortality. Recently, the disease caused by FAdV-4 has spread globally and lead to substantial economic losses to the poultry industry (3). Therefore, many vaccines or vaccine candidates including inactivated vaccines and sub-unit vaccines have been developed to control the disease (4). However, few live-attenuated vaccines against FAdV-4 were available.

It is well-known that the Fiber protein of FAdV plays a vital role in the infection and pathogenicity. Different from most serotypes of FAdV, FAdV-4 has two Fiber proteins, designated as Fiber-1 and Fiber-2 (5). Recently, Fiber-2 was identified to be closely related with the virulence of FAdV-4, and the *fiber-2*-edited recombinant viruses FA4-EGFP and FAV-4_Del were highly attenuated to the SPF chickens (6–8). Notably, Fiber-1, but not Fiber-2, was identified to directly trigger viral infection via its knob and shaft domain through the cellular receptor, the CAR homology (9–11). To investigate whether the *fiber-1* could be further edited to develop FAdV-4 live-attenuated vaccine or vector, we targeted the region between the tail and shaft of Fiber-1 at the 87th amino acid of its N-terminus to generate a recombinant virus FAdV4-RFP_F1 with the expression of the fusion protein RFP (The Red Fluorescence Protein) and Fiber-1 through the CRISPR/Cas9 technique. *In vitro* and *in vivo* studies revealed that the rescued FAdV4-RFP_F1 was not only highly attenuated, but could also provide efficient protection against the lethal challenge.

## Results

### Generation of a Novel Recombinant Virus FAdV4-RFP_F1

**Figure 1:**
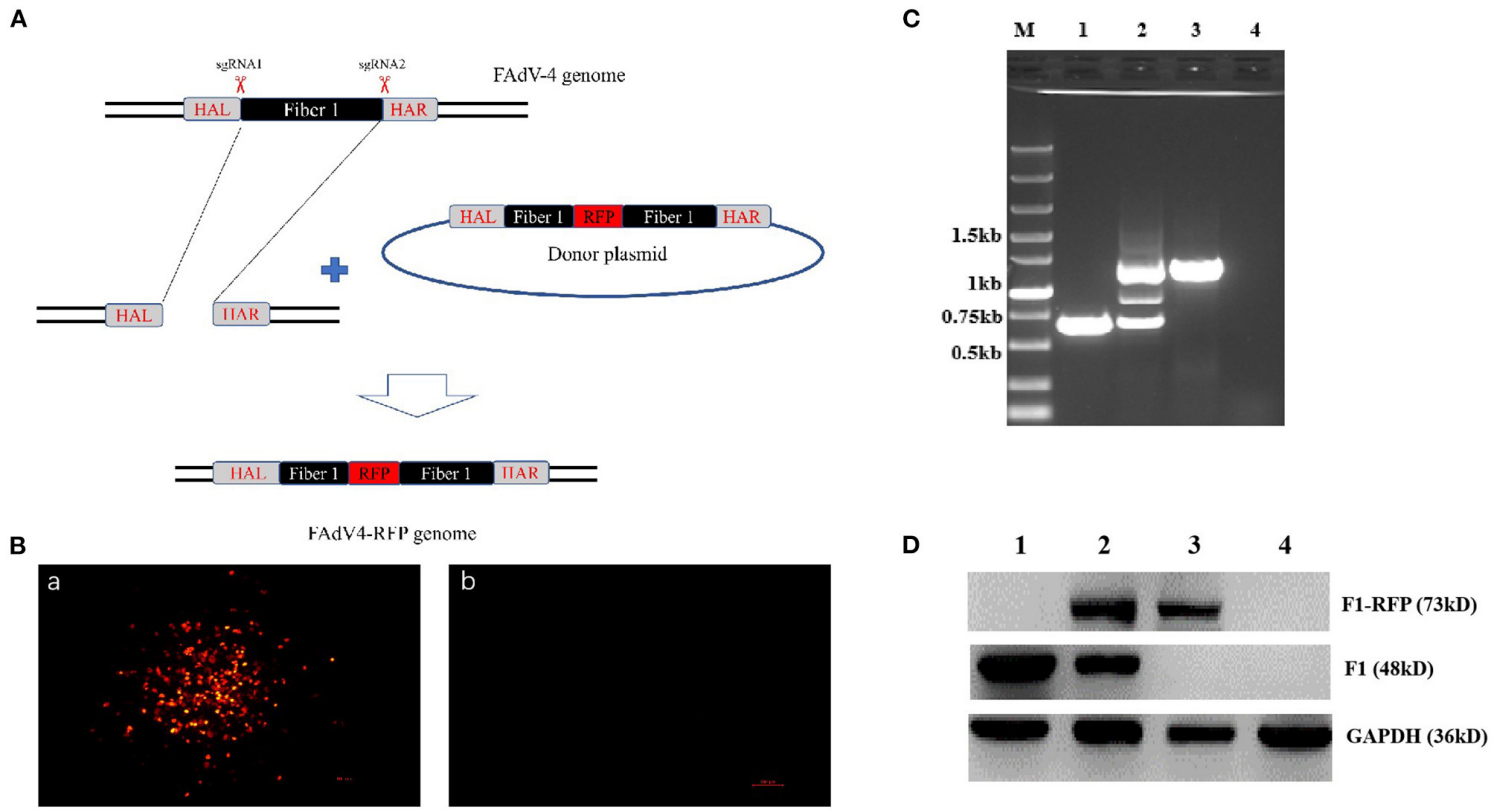
Generation and identification of a novel recombinant virus FAdV4-RFP_F1. **(A)** Strategy of the CRISPR/Cas 9 system for generating the recombinant virus FAdV4-RFP_F1. LMH cells were transfected with sgRNA targeting the *fiber-1* gene. At 24 hpi, the LMH cells were inoculated with FAdV-4 and transfected with donor plasmid. Then the recombinant virus FAdV4-RFP_F1 was purified by limiting dilution assay and viral plaque assay. **(B)** The RFP could be found by fluorescence microscopy in LMH cells transfected with sgRNA and donor plasmid (a), but not be found in LMH cells without infection (b). **(C)** PCR identification of the recombinant virus FAdV4-RFP_F1. The WT FAdV-4 (lane 1), unpurified recombinant virus FAdV4-RFP_F1 (lane 2), and the purified FAdV4-RFP_F1 (lane 3) were detected using specific primers. **(D)** Western blot analysis of the recombinant virus FAdV4-RFP_F1. The LMH cells infected with the WT FAdV-4 (lane 1). The unpurified recombinant FAdV4-RFP_F1 (lane 2) and the purified FAdV4-RFP_F1 (lane 3) were harvested and lysed, and the lysates were then tested with Western blot by chicken sera against FAdV-4 Fiber-1.

To explore whether the *fiber-1* could be edited to develop FAdV-4 live-attenuated vaccine or vector, two sgRNAs targeting the *fiber-1* of FAdV-4 were designed and cloned into CRISPR/Cas9 vector, and subsequently transfected into LMH cells, followed with the transfection of the donor plasmid and the infection of FAdV-4 as shown in Figure 1A. After viral infection, RFP could be efficiently found in LMH cells transfected with sgRNA and donor plasmid, but no RFP could be found in LMH cells without infection (Figure 1B), indicating that the recombinant virus, designated as FAdV4-RFP_F1, was successfully generated. After purification by limiting dilution and plaque assay, the purified virus was confirmed by PCR, sequencing, and western blot. As described in Figure 1C, the specific single band of RFP-fiber-1 could be amplified from the purified FAdV4-RFP_F1 whereas two bands could be amplified from the unpurified FAdV4-RFP_F1 by the specific primers listed in **Table 2**. Further identification with sequencing showed that the recombinant virus FAdV4-RFP_F1 was constructed exactly as designed (data not shown). In addition, western blot assay showed that the specific band of RFP-Fiber-1 fusion protein could be efficiently detected in the LMH cells infected with the purified FAdV4-RFP_F1 as shown in Figure 1D. Taken together, all these data demonstrate that the recombinant virus FAdV4-RFP_F1 expressing RFP-Fiber-1 fusion protein is successfully generated.

### Efficient Replication and High Stability of FAdV4-RFP_F1 *in vitro*

**Figure 2:**
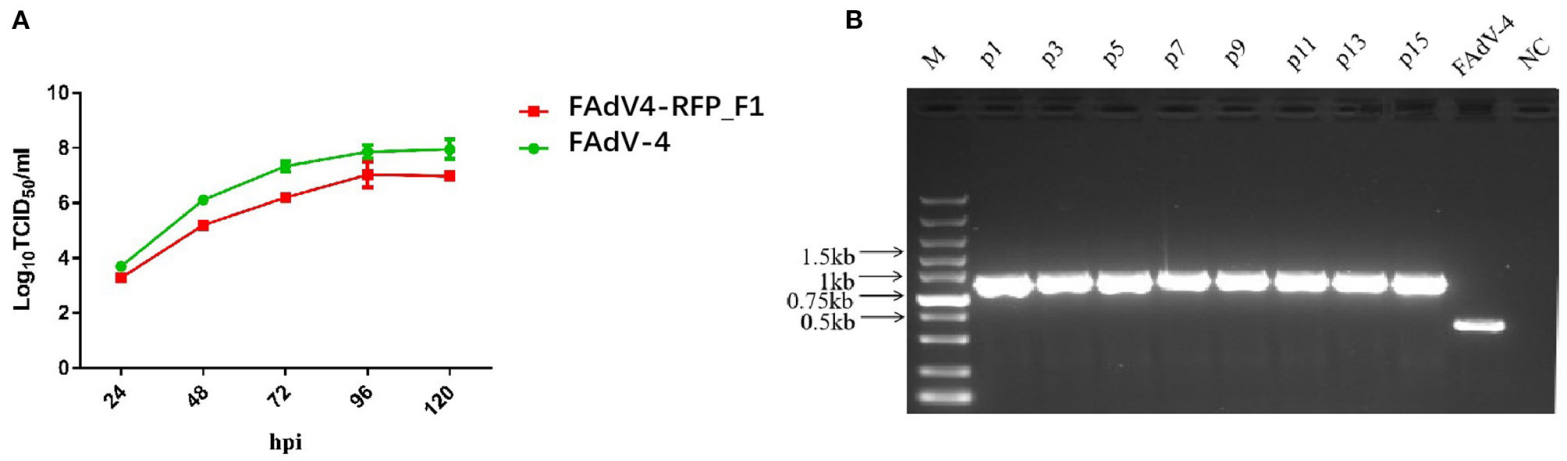
Growth kinetics and stability of FAdV4-RFP_F1 *in vitro*. **(A)** LMH cells were inoculated with FAdV4-RFP_F1 and WT FAdV-4 at the same dose, then the virus-containing supernatants were collected at the indicated time points for virus titration. **(B)** The virus FAdV4-RFP_F1 was serially passaged for 15 generations with the dilution of 1:10,000 for each generation, and then the viral supernatants were collected and detected by PCR.

To investigate the growth characteristics of the recombinant virus *in vitro*, FAdV4-RFP_F1 and WT FAdV-4 were inoculated into LMH cells at the same dose and the viral supernatants were collected at different time points and determined by TCID_50_. As shown in Figure 2A, the recombinant virus FAdV4-RFP_F1 with *fiber-1-*edited could still replicate efficiently in LMH cells. Although the FAdV4-RFP_F1 replicated slower than the WT FAdV-4, the peak viral titer of FAdV4-RFP_F1 could still reach 10^7.0^ TCID_50_/ml at 5 dpi. Moreover, to test the stability of the recombinant virus, the FAdV4-RFP_F1 was serially passaged with 15 generations in LMH cells, and then the viral supernatants were collected and examined by PCR. As shown in Figure 2B, the unique band of the RFP-fiber-1 could be efficiently amplified by the specific primers whereas the band specific to the WT FAdV-4 could not be amplified in each generation of FAdV4-RFP_F1 tested. All these reveal that the FAdV4-RFP_F1 can efficiently and stably replicate in LMH cells.

### FAdV4-RFP_F1 Was Highly Attenuated *in vivo*

**Figure 3:**
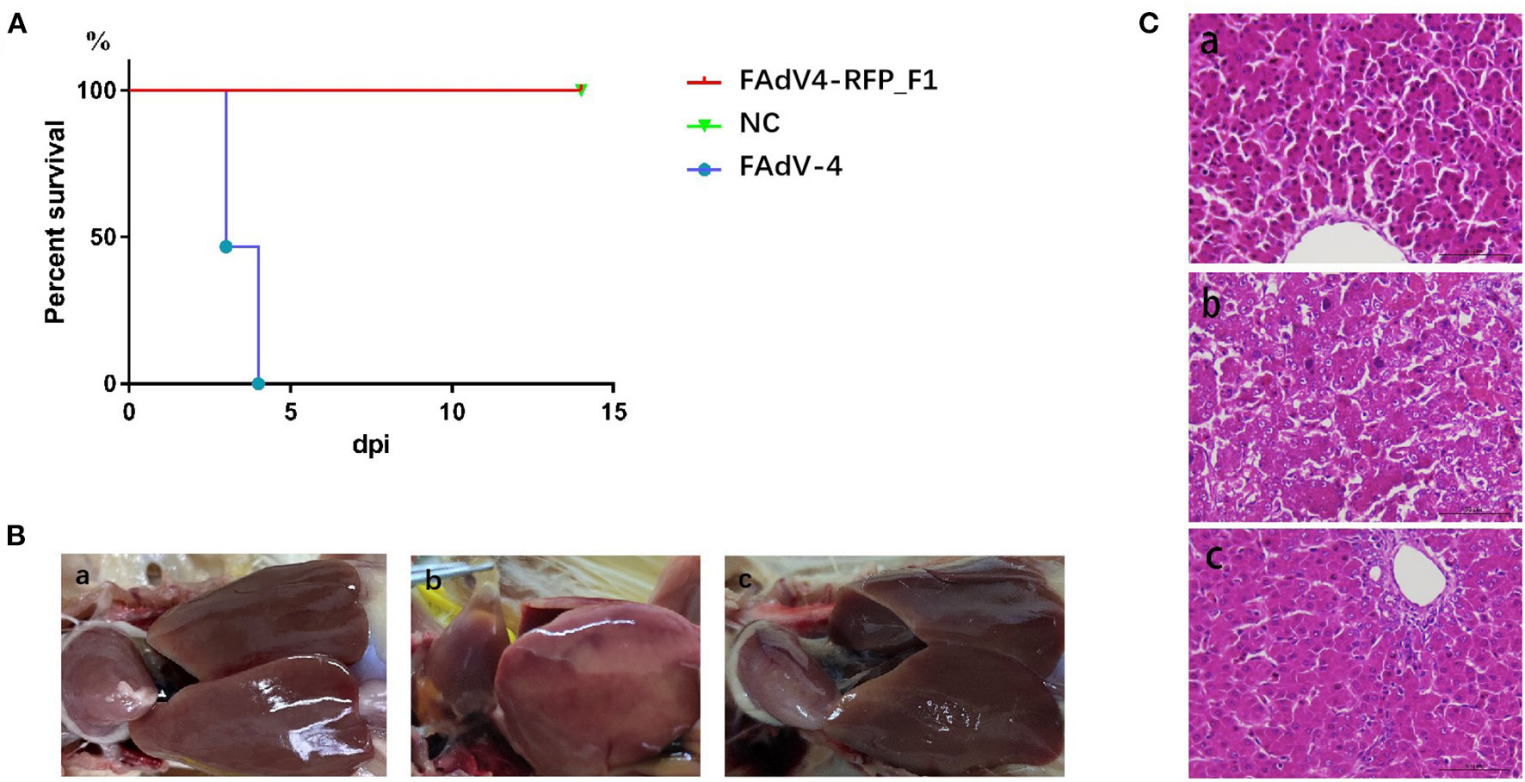
FAdV4-RFP_F1 was highly attenuated *in vivo*. All the SPF chickens were randomly divided into three groups and inoculated with FAdV-4, FAdV4-RFP_F1, and 1% culture medium, respectively. All the chickens were monitored for 14 days. **(A)** Percent of survival for the infected chickens. **(B)** Pathological changes in the chickens from the chickens inoculated with FAdV4-RFP_F1 (a), FAdV-4 (b), and the negative control group (c). **(C)** Representative histological changes in liver tissues from the chickens inoculated with FAdV4-RFP_F1 (a), FAdV-4 (b), and the negative control group (c).

**Figure 4:**
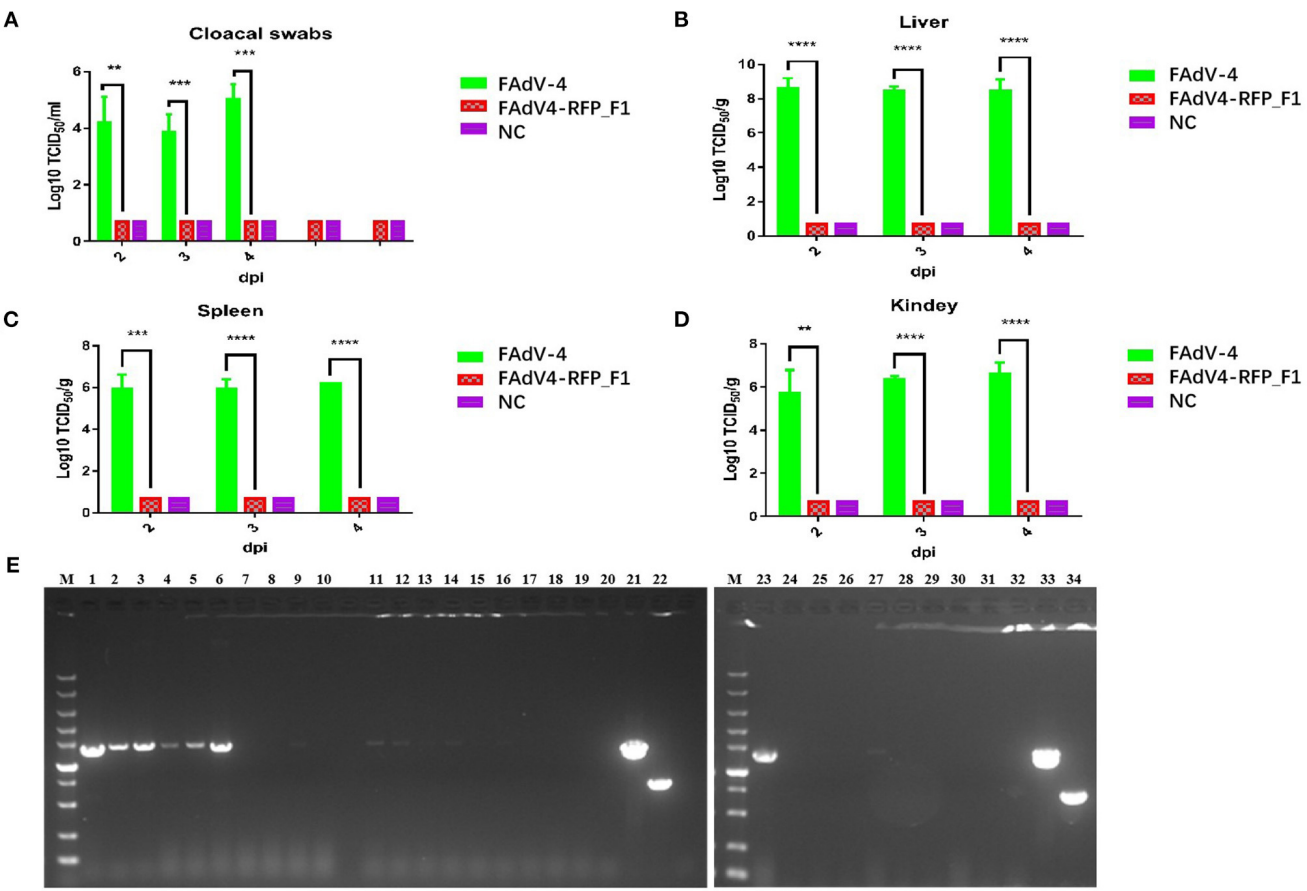
Viralsheddingin cloaca andload in tissues from the infected chickens. **(A)** Viral shedding in cloacal swabs from the infected chickens. Viral loads in liver **(B)**, spleen **(C)**, and kidney **(D)** from the infected chickens. **(E)** PCR identification of tissues from the chickens infected with FAdV4-RFP_F1. Liver samples (2 dpi: lane 1–3; 3 dpi: lane 4–6; 4 dpi: lane 7–9); spleen samples (2 dpi: lane 11–13; 3 dpi: lane14–16; 4 dpi: lane 17–19); kidney samples (2 dpi: lane 23–25; 3 dpi: lane 26–28; 4 dpi: lane 29–31). PCR identification of tissues from the chickens of the negative control group (liver sample: lane 10; spleen sample: lane 20; kidney sample: lane 32). Cell supernatant of FAdV4-RFP_F1 (lane 21 and 33); cell supernatant of FAdV-4 (lane 22 and 34). ^**^, ^***^, and ^****^ indicate *P*-value < 0.05, 0.01, 0.001, and 0.0001, respectively.

To evaluate the replication and pathogenicity of the recombinant virus FAdV4-RFP_F1 *in vivo*, SPF chickens were infected with FAdV4-RFP_F1 and WT FAdV-4 virus at the same dose. The chickens inoculated with F12-DMEM containing 1% of FBS were set as negative control. The clinical symptoms and mortality of the inoculated chickens were monitored daily. The cloacal swabs and organs from different groups were collected and analyzed at the indicated time points. The chickens infected with FAdV-4 exhibited signs of depression, loss of appetite, lethargy, and yellow-green and thin feces at 2 dpi. Fifty-three percent and 100% of the chickens died at 3 dpi and 4 dpi, respectively. In contrast, chickens infected with FAdV4-RFP_F1 were all survived and did not show any clinical symptoms as described in Figure 3A. Moreover, the typical lesion, hepatitis-hydropericardium syndrome, could be observed in the chickens infected with FAdV-4 after necropsy analysis, while that in the other two groups could not be found (Figure 3B). In addition, the histopathological analysis demonstrated that chickens in the FAdV-4 group presented sever IBH in liver tissues, whereas these in FAdV4-RFP_F1 group were similar with the negative control group (Figure 3C). According to the viral shedding in the cloaca, the viral titers of the chickens infected with FAdV-4 could reach up to 10^5^ TCID_50_/ml at 2–5 dpi, whereas no virus could be detected in the chickens infected with FAdV4-RFP_F1 during 2–8 dpi (Figure 4A). For the virus tissue load, the high level of viral titer could be detected in the livers, spleens, and kidneys in the chickens infected with FAdV-4 at 2–4 dpi. Of note, the viral titer in the livers in the chickens infected with FAdV-4 could reach up to 10^8^ TCID_50_/ml, followed by kidney and spleen, whereas no virus could be detected in the chickens infected with FAdV4-RFP_F1 by TCID_50_ as shown in Figures 4B–D. However, the recombinant virus FAdV4-RFP_F1 in the organic tissues could be detected by PCR at early time points (Figure 4E). Taken together, all these demonstrate that the FAdV4-RFP_F1 is highly attenuated *in vivo*.

### FAdV4-RFP_F1 Provided Efficient Protection Against Lethal Challenge

**Figure 5:**
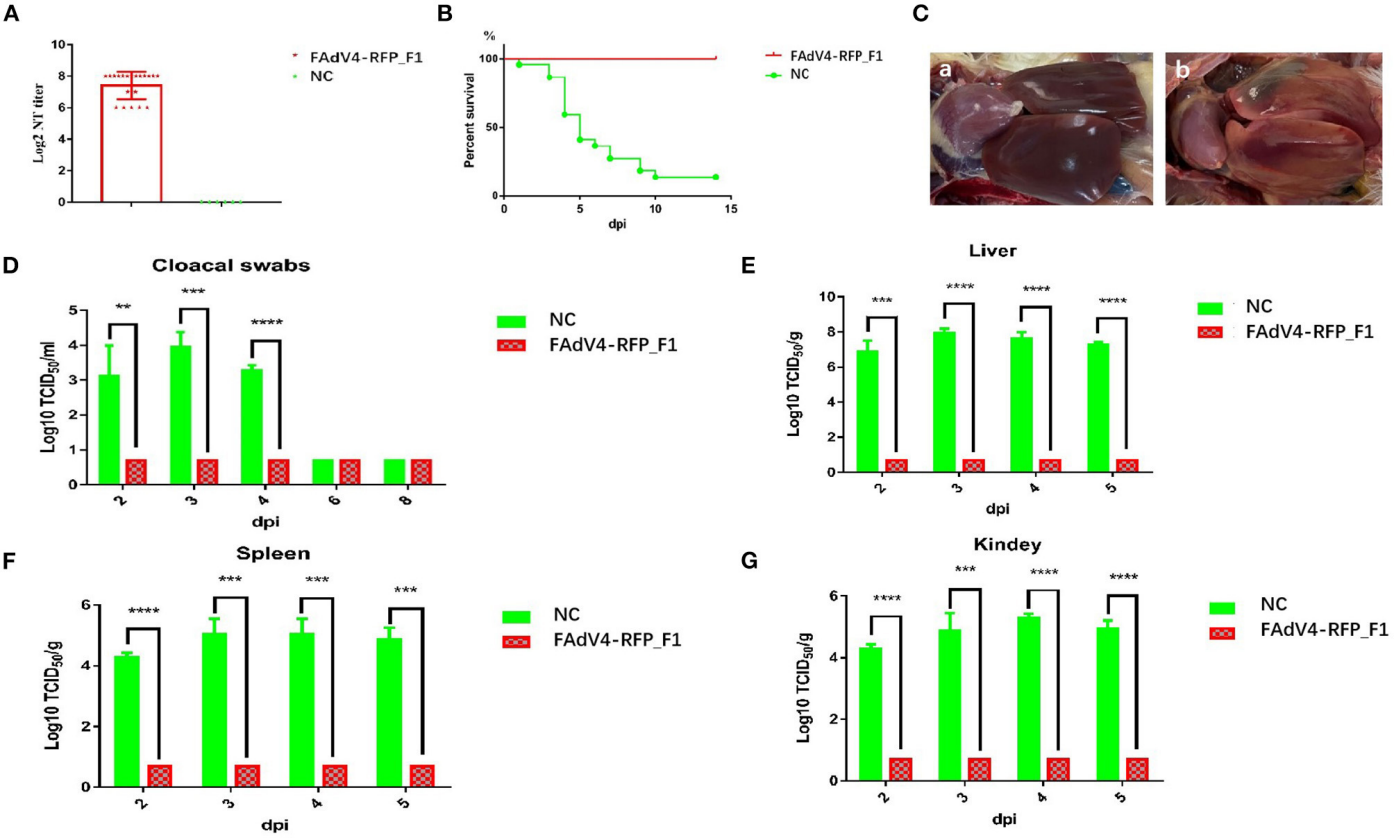
FAdV4-RFP_F1 provided strong protection against the lethal challenge. The survival chickens inoculated with FAdV4-RFP_F1 and 1% culture medium were challenged at 21 dpi with the lethal dose of FAdV-4. **(A)** Neutralizing antibody titer in chickens inoculated with FAdV4-RFP_F1 at 21 dpi. **(B)** Percent of survival for the challenged chickens. **(C)** Pathological changes from the challenged chickens previously inoculated with FAdV4-RFP_F1 (a) and the challenged control chickens (b). **(D)** Viral loads in cloacal swabs from the challenged chickens. Viral loads in liver **(E)**, spleen **(F)**, and kidney **(G)** from the challenged chickens. ^**^, ^***^, and ^****^ indicate *P*-value < 0.05, 0.01, 0.001, and 0.0001, respectively.

To evaluate whether the recombinant virus FAdV4-RFP_F1 could provide protective efficacy against FAdV-4, the sera from chickens in the control group or infected with FAdV4-RFP_F1 were first collected at 21 dpi for the detection of neutralizing antibody, and all these chickens were then challenged with the lethal dose of the WT FAdV-4 at 21 dpi. The clinical symptoms and mortality of the chickens were monitored daily. The cloacal swab and tissue samples from chickens were collected at the indicated time points and titrated for viral titers. As described in Figure 5A, the chickens infected with FAdV4-RFP_F1 at 21 dpi could produce high level of neutralizing antibody with the average titer of about 2^7.4^ whereas the neutralizing antibody could not be detected in the control group. After challenge, the chickens in the control group showed clinical symptom and the mortality rate reached 82% (19/23) at 10 dpi, whereas the chickens previously infected with FAdV4-RFP_F1 did not show any clinical symptoms or death through the animal experiment as shown in Figure 5B. In the necropsy analysis, the typical lesion, hepatitis-hydropericardium syndrome was observed in the chickens from the control group (Figure 5C). Moreover, the high level of viral titers in the cloacal swab and tissue samples from the chickens of the control group were efficiently detected, whereas those from chickens previously infected with FAdV4-RFP_F1 could be not detected (Figures 5D–G). All these data clearly demonstrate that the recombinant virus FAdV4-RFP_F1 can provide efficient protection against the lethal challenge of FAdV-4.

## Discussion

Hepatitis-hydropericardium syndrome caused by the highly pathogenic FAdV-4 has resulted in substantial economic losses to poultry industry globally. To control the disease, the vaccination is one of the efficient strategies (12). Up to now, various vaccines or vaccine candidates have been generated, including the inactivated vaccines, the recombinant subunit vaccines, and the live-attenuated vaccines (4, 13–17). Generally, different vaccines have different advantages or disadvantages. The inactivated whole virus vaccines can provide efficient protection, but these vaccines are generally costly. Although the Fiber-2 subunit vaccines are also widely used, Fiber-2 cannot induce detectable neutralizing antibodies to completely prevent the viral shedding. The live-attenuated vaccines can both induce strong humoral and cellular immune responses and show a great promise for efficiently controlling the diseases caused by FAdV-4. Currently, the live-attenuated vaccines against FAdV-4 were mainly generated through cell or host adaptation of the virulent strains (4). Since the altered or adapted genomes of such live-attenuated vaccines are generally uncontrolled, live-attenuated vaccines against FAdV-4 with specific gene-edited are badly needed.

Recently, we used CRISPR-Cas9 technique to target the virulent gene *fiber-2* to generate two live-attenuated *fiber-2*-edited vaccine candidates FA4-EGFP and FAV-4_Del (7, 8). In this study, we targeted another structural protein Fiber-1 of FAdV-4 to rescue a novel recombinant virus FAdV4-RFP_F1 expressing the fusion protein of RFP and Fiber-1. Since our previous study demonstrated that the shaft and knob domains of Fiber-1 play vital roles in directly triggering the viral infection of FAdV-4 (10), the inserted site of RFP and the sgRNA target were designed at the region between the tail and shaft of Fiber-1 at the 87th amino acid of its N-terminus. Of note, the rescued FAdV4-RFP_F1 not only replicated efficiently and stably *in vitro*, but was also avirulent *in vivo*. These indicate that although the Fiber-1 plays a critical role in triggering the infection of FAdV-4, the N-terminal domain of Fiber-1 can be an efficient target to be edited to generate recombinant viruses for expressing foreign genes. Moreover, the recombinant virus FAdV4-RFP_F1 did not shed in the cloaca and showed limited replication in the tissues tested in the inoculated chickens, but it could provide efficient protection against the lethal challenge of the WT FAdV-4, highlighting FAdV4-RFP_F1 generated here can be as an efficient live-attenuated vaccine for preventing the diseases caused by FAdV-4.

It should be noted that although the recombinant virus FAdV4-RFP_F1 could replicate efficiently in LMH cells, but it could not efficiently replicate in liver, spleen, and kidney in the infected chickens through intramuscular injection. Whether the recombinant virus FAdV4-RFP_F1 with the edited *fiber-1* can alter the tissue tropism *in vivo* needs to be elucidated. Recently, although Zhang et al. showed that the knob of Fiber-1 could also be edited to insert the exogenous peptide, several generated recombinant viruses could alter the cell tropism *in vitro* (18). To investigate the mechanism for the protective efficacy for FAdV4-RFP_F1 against FAdV-4, the sera from chickens before challenge were collected and tested for neutralizing antibody. It was unexpected that these chickens inoculated with FAdV4-RFP_F1 at 21 dpi could produce high level of neutralizing antibody with the average titer of about 2^7.4^ (Figure 5A). Wu et al. found that the expression of the anti-inflammatory cytokines IL-4, IL-10, and transforming growth factor (TGF)-β1 and TGF-β2 were upregulated in FAdV-4-infected chickens (19) and the infection of FAdV-4 could induce strong innate immune responses in chickens (20). Therefore, except for the contribution of the humoral immune response, the innate and cellular immune response induced by FAdV4-RFP_F1 in the inoculated chickens should play vital roles in the protection, which need to be further clarified.

In summary, this is the first demonstration of a novel *fiber-1*-edited and highly attenuated recombinant virus FAdV4-RFP_F1. Notably, FAdV4-RFP_F1 could efficiently replicate *in vitro*, but not *in vivo*. However, FAdV4-RFP_F1 could provide strong protection against the lethal challenge of FAdV-4. The generation of the avirulent recombinant virus FAdV4-RFP_F1 carrying the fusion protein of RFP and Fiber-1 with high viral titer highlights FAdV4-RFP_F1 can be an efficient live-attenuated vaccine against FAdV-4, and the N-terminal domain of *fiber-1* can be edited as a potential insertion site for expressing foreign genes. The cellular immune protection induced by the live-attenuated recombinant virus FAdV4-RFP_F1 needs to be further elucidated. Since the co-infection could significantly promote the pathogenicity of FAdV-4 and inhibit the immune response in chickens (21), whether the RFP in FAdV4-RFP_F1 can be replaced with other protective antigens to develop FAdV-4 based multiple vaccines both for controlling of FAdV-4 and other poultry pathogens also need to be evaluated.

## Materials and Methods

### Cells, Viruses, and Antibodies

Chicken hepatoma cells (Leghorn male hepatoma, LMH) were purchased from American Type Culture Collection (Manassas, VA, USA) and cultured in F12-Dulbecco's Modified Eagle Medium (DMEM; Gibco, NY, USA) supplemented with 10% fetal bovine serum (FBS; Lonsera, Shanghai, China) in a 5% CO_2_ incubator at 37°C, and passaged every 3 days. The FAdV-4 strain SD2015 was isolated and stored in our laboratory. The monoclonal antibody (mAb) 3C2 against Fiber-2 of FAdV-4 and chicken sera against FAdV-4 were prepared in our laboratory (22).

### Construction of sgRNA and Donor Plasmids

The sgRNA targeting the *fiber-1* of FAdV-4 was designed according to the CRISPR guide RNA designing website (www.benchling.com) and cloned into the CRISPR/Cas9 vector lentiCRISPR v2 (23). The sequences of the sgRNA were listed in Table 1. The donor plasmid with the RFP gene located after the N-terminal 87 amino acid of the *fiber-*1 was constructed by overlap extension PCR and the homologous arm (HA) was designed in 1,000 bp length at both ends, respectively. The template was assembled as the HAL-F1-RFP-F1-HAR and then cloned into the pMD19 simple vector. The primers used for constructing donor plasmid were shown in Table 2.

**Table 1:** Primers used for the construction of sgRNA.

	**Sequence of primers (5^**′**^ → 3^**′**^)**
sgRNA1	F1-sgRNA1_F: CACCGACCTAGCCGGTACCTTTCGG
	F1-sgRNA1_R: AAACCCGAAAGGTACCGGCTAGGTC
sgRNA2	F1-sgRNA2_F: CACCGGGTTACGTCTACTCCCCCAA
	F1-sgRNA2_R: AAACTTGGGGGAGTAGACGTAACCC

**Table 2:** Primers used for the construction of donor plasmid and the identification of the recombinant virus.

**PCR products**	**Sequence of primers (5^**′**^ → 3^**′**^)**
RFP	F: CCCATCGGGGTCGACCGCGATCTG
	R: CCCATCGGGGTCGACCGCGATCTG
HAL+Fiber-1	F: GACCGGGGACATTTATACTGTC
	R: CCCATCGGGGTCGACCGCGATCTG
Fiber-1+ HAR	F: CCCATCGGGGTCGACCGCGATCTG
	R: CCCATCGGGGTCGACCGCGATCTG
Partial Fiber-1	F: CTGACGGTAATCGGTCTCGG
	R: CTGTGAGTCGACGAGCACTT

### Rescuing of the Recombinant Virus FAdV4-RFP_F1

LMH cells seeded in 6-well plate were transfected with sgRNAs targeting the *fiber-1* gene at 2 μg of each. At 24-h post-transfection (hpt), the LMH cells were inoculated with FAdV-4 at a multiplicity of infection (MOI) of 0.1 and immediately transfected with the donor plasmid at 4 μg. The rescued recombinant virus, designated as FAdV4-RFP_F1, was observed through a fluorescence microscopy and further purified by limiting the dilution assay and virus plaque assay. The purified virus was then identified by PCR, sequencing, and western blot.

### Growth Curve of the Recombinant Virus FAdV4-RFP_F1

The recombinant virus FAdV4-RFP_F1 and wild type FAdV-4 were inoculated into LMH cells at 0.1 MOI, respectively. At 2 h post-infection (hpi), the cells were washed with PBS and maintained with the fresh F12-DMEM containing 1% of FBS. Then, the viral supernatants were harvested at 24, 48, 72, 96, and 120 dpi, and then titrated in LMH cells by IFA. The TCID_50_ of the viruses were calculated by the Reed–Muench method (24). The final viral growth curves were constructed with GraphPad Prism 7 software.

### Stability of the Recombinant Virus FAdV4-RFP_F1

To test the stability, the purified recombinant virus was serially passaged in LMH cell with the dilution of 1:10,000 for each generation, and the supernatants were collected from each generation and examined by PCR using specific primers.

### Western Blot Assay

LMH cells either infected with the recombinant virus FAdV4-RFP_F1 or wild type FAdV-4 were lysed in lysis buffer (CST, MA, USA) with phenylmethylsulfonyl fluoride (PMSF; Beyotime, Shanghai, China), protease, and phosphatase inhibitors (CST, MA, USA). The lysates were then boiled in the loading buffer. After subjected to 10% SDS-PAGE, the denatured proteins were transferred to nitrocellulose (NC) membranes (GE Healthcare Life sciences, Freiburg, Germany). After blocking with 5% skimmed milk in PBS with Tween 20 (PBST) for 2 h at room temperature (RT), the membranes were reacted with the corresponding mAbs or sera for 2 h at RT. After three washes with PBST, the membranes were incubated with horseradish peroxidase (HRP)-labeled secondary antibodies for 1 h at RT. After another three washes, the membranes were developed with chemiluminescent reagents and imaged with an automatic imaging system (Tanon 5200).

### Indirect Immunofluorescent Assay

The LMH cells infected with viruses were fixed with the ice-cold acetone–ethanol (3:2) mixture for 5 min at RT and washed with PBS. The fixed cells were then incubated with the diluted mAb 3C2 against Fiber-2 for 45 min at 37°C. After washing three times with PBS, the cells were incubated with the diluted secondary antibody (goat anti-mouse antibody labeled with fluorescein isothiocyanate, FITC) for another 45 min at 37°C. Again, after three washes with PBS, the cells were observed by invert fluorescence microscopy.

### Neutralization Test

Different dilutions of the chicken sera were first mixed with 200 TCID_50_ of FAdV-4 and incubated for 1 h at 37°C. Then, the mixtures were added to the 96-well plate with LMH cells and incubated for 2 h at 37°C. After washing once, the cells were cultured in F12-DMEM with 1% fetal bovine serum. After culturing for 96 h, the cells were fixed and subjected to IFA analysis by using mAb 3C2 against Fiber-2 as previously described (22).

### Animal Experiment

A total of 102 2-week-old specific pathogen free (SPF) chickens were randomly divided into three groups (chickens in group I (*n* = 34) were infected with FAdV-4 as a positive control group; chickens in group II (*n* = 34) were infected with FAdV4-RFP_F1 as experimental group; and chickens in group III (*n* = 34) without infection was set as negative control) and inoculated through intramuscular injection. The chickens in group I and II were inoculated at a dose of 2 × 10^5^ TCID_50_ in 200 μl of 1% culture medium, whereas the chickens in group III were inoculated with the same volume of 1% culture medium. The cloacal swabs of each group were collected at indicated time points post-infection. Three chickens in each group were euthanized at 2, 3, and 4 dpi. The livers, spleens, and kidneys of these chickens were collected for viral titration or histopathological analysis. At 21 dpi, chickens that survived in group II and group III were challenged with 2 × 10^6^ TCID_50_ of FAdV-4 in 200 μl of 1% culture medium via intramuscular injection to further evaluate the protective efficacy. The cloacal swabs were collected at indicated time points post-challenge (pc). Three chickens in each group were euthanized at 2, 3, 4, and 5 dpc. The livers, spleens, and kidneys of these chickens were collected for viral titration. The clinical symptoms and mortality of the inoculated chickens were monitored daily.

### Titration of Viral Titer in the Organs and Cloacal Swab Samples

The homogenates of liver, spleen, and kidney from the chickens were treated with 10× penicillin and streptomycin for 1 h at 37°C and centrifuged to obtain the supernatant. The cloacal samples collected from the chickens were placed in 800 μl of PBS. After three times of repeated freeze–thaw cycles, the samples were also treated with 10× penicillin and streptomycin for 1 h at 37°C and centrifuged to obtain the supernatant. The virus-containing supernatants were serially diluted in F12-DMEM with 1% of FBS and were inoculated into the 96-well plates with LMH cells. The infected LMH cells were then fixed and detected by IFA using mAb 3C2 against Fiber-2 at 4 dpi, and TCID_50_ of those supernatants were calculated by the Reed–Muench method.

### Statistical Analysis

All the results are presented as means ± standard deviation. The statistical analysis in this study was performed with the Student's *t*-test using GraphPad 7 software. *P*-value of < 0.05 was considered significant. ^*^, ^**^, ^***^, and ^****^ indicate *P*-value < 0.05, 0.01, 0.001, and 0.0001, respectively.

## Data Availability Statement

The original contributions presented in the study are included in the article/supplementary material, further inquiries can be directed to the corresponding author/s.

## Ethics Statement

The animal study was reviewed and approved by the Animal Care Committee at Yangzhou University in China.

## Author Contributions

JY and AQ designed the project. YM, QX, WW, HL, ML, ZW, and TL carried out the experiment. YM, QX, HS, ZW, and WG analyzed the data. YM, QX, and JY drafted the manuscript. JY supervised all the experiments and participated in the data analysis. YM, QX, ZW, HS, and AQ discussed and prepared the final reported. All of the authors have read and approved the final manuscript.

## Funding

This study was supported by Jiangsu Agricultural Science and Technology Innovation Fund (CX(19)3026), the Key Research & Development (R&D) Plan in Yangzhou City (YZ2020052), Key Laboratory of Prevention and Control of Biological Hazard Factors (Animal Origin) for Agrifood Safety and Quality (26116120), and Research Foundation for Talented Scholars in Yangzhou University and the Priority Academic Program Development of Jiangsu Higher Education Institutions.

## Conflict of Interest

The authors declare that the research was conducted in the absence of any commercial or financial relationships that could be construed as a potential conflict of interest.

## Publisher's Note

All claims expressed in this article are solely those of the authors and do not necessarily represent those of their affiliated organizations, or those of the publisher, the editors and the reviewers. Any product that may be evaluated in this article, or claim that may be made by its manufacturer, is not guaranteed or endorsed by the publisher.
